# Attentional Bias towards Positive Emotion Predicts Stress Resilience

**DOI:** 10.1371/journal.pone.0148368

**Published:** 2016-03-23

**Authors:** Hanna A. Thoern, Marcus Grueschow, Ulrike Ehlert, Christian C. Ruff, Birgit Kleim

**Affiliations:** 1 Department of Clinical Psychology and Psychotherapy, University of Zurich, Zurich, Switzerland; 2 Laboratory for Social and Neural Systems Research, Department of Economics, University of Zurich, Zurich, Switzerland; 3 Department of Psychiatry, Psychotherapy and Psychosomatics, University of Zurich, Zurich, Switzerland; 4 Department of Experimental Psychopathology and Psychotherapy, University of Zurich, Zurich, Switzerland; University of Verona, ITALY

## Abstract

There is extensive evidence for an association between an attentional bias towards emotionally negative stimuli and vulnerability to stress-related psychopathology. Less is known about whether selective attention towards emotionally *positive* stimuli relates to mental health and stress resilience. The current study used a modified Dot Probe task to investigate if individual differences in attentional biases towards either happy or angry emotional stimuli, or an interaction between these biases, are related to self-reported trait stress resilience. In a nonclinical sample (*N* = 43), we indexed attentional biases as individual differences in reaction time for stimuli preceded by either happy or angry (compared to neutral) face stimuli. Participants with greater attentional bias towards happy faces (but not angry faces) reported higher trait resilience. However, an attentional bias towards angry stimuli moderated this effect: The attentional bias towards happy faces was only predictive for resilience in those individuals who also endorsed an attentional bias towards angry stimuli. An attentional bias towards positive emotional stimuli may thus be a protective factor contributing to stress resilience, specifically in those individuals who also endorse an attentional bias towards negative emotional stimuli. Our findings therefore suggest a novel target for prevention and treatment interventions addressing stress-related psychopathology.

## Introduction

The disrupting effect of negative emotional stimuli on attention has been firmly associated with anxiety disorders [1,2]. There is indeed ample evidence for an association between an attentional bias (AB) to threatening stimuli and the development and maintenance of anxiety [1–4]. Compared to non-anxious individuals, anxious individuals show a preference to attend to threatening over non-threatening material. In stark contrast to this ever-expanding body of literature on attention to negative or threatening material, selective attention to positive material has received considerably less attention. Just like an AB to negative information may cause vulnerability to anxiety, an AB to positive information may play an important role in determining mental health and stress resilience, i.e. adaptive psychological and physiological responding to stressors (e.g., [5]). More specifically, an AB towards positive stimuli may facilitate adaptive stress regulation by preventing negative emotional responses to stressors and increase reward direction (e.g., [6]). Endorsing an AB to positive emotional stimuli may thus constitute an adaptive part of antecedent emotion regulation that operates before an emotional response is fully generated and thus leads to resilient responding via the experience of positive emotions.

Some studies have investigated AB to positive stimuli in the context of psychopathology and suggest that an AB to positive or reward-related stimuli is related to low levels of anxiety or the absence of psychopathology [2]. Experimentally, participants with higher AB towards positive stimuli showed mitigated stress responses and endured longer on a stressful anagram task [7]. In a prospective study, AB towards positive stimuli predicted less subjective stress to a laboratory stressor four months later [8]. Interestingly, an AB away from negative stimuli also predicted reduced stress in response to both laboratory [8] and naturalistic stressors [8–10]. All of these studies indexed absence of depression, anxiety or negative emotion as key outcomes and did not specifically assess stress resilience. Moreover, most of the studies investigate separate influences of AB towards positive and negative stimuli, without taking into account the possibility that these biases may interact (e.g., [11]). Based on findings that AB towards negative information is attenuated when positive constructs are made accessible [12], it stands to reason whether the impact of AB towards positive stimuli is modulated by a negative attentional bias and the accessibility of negative material. This would mean that only those individuals who endorse a negative AB may require and display a positive AB along with its beneficial effects. Statistically, such an effect would be evident if an AB towards negative material would moderate the effect of AB towards positive material on resilience.

The aim of the current study was to investigate the influence of AB to positive and negative emotional material in the context of stress resilience. AB was indexed with a modified version of the frequently used Dot Probe Task employing positive, negative and neutral facial stimuli (DPT; [13]). Based on the empirical findings presented above, we hypothesized that (i) there are individual differences in expression of an AB towards positive face stimuli and that greater AB to positive stimuli would predict greater trait stress resilience. We also explored whether (ii) an AB away from negative face stimuli would predict trait stress resilience and (iii) whether an AB towards negative stimuli moderates the association between AB towards positive stimuli and trait resilience. Understanding factors that promote resilience in the face of stress is crucial for the development of stress prevention programs and for improving treatment of stress-related disorders.

## Method

### Participants

Forty-three (11 male, 32 female) healthy adults participated in exchange for 25 Swiss Francs (27 US$). All participants were right-handed and had normal or corrected-to-normal vision. The mean age of participants was 27 years (*SD* = 6.50). The local ethic board (i.e., Kantonale Ethikkommission Zürich) approved the study and all participants gave their written informed consent prior to participating in the study.

### Measures

#### Self-reported trait stress resilience

Stress resilience was measured with the German version of the Resilience Scale (RS-11; [14,15]). The RS-11 comprises 11 items ranging from 1–7, and answers are added up to a sum score indexing trait resilience, with higher scores reflecting greater trait resilience. The RS-11 has high internal consistency (Cronbach *α* = .91), and has shown convergent validity with related constructs. Internal consistency was high in the present sample, *α* = .84.

#### Modified Dot Probe Task

**Fig 1:**
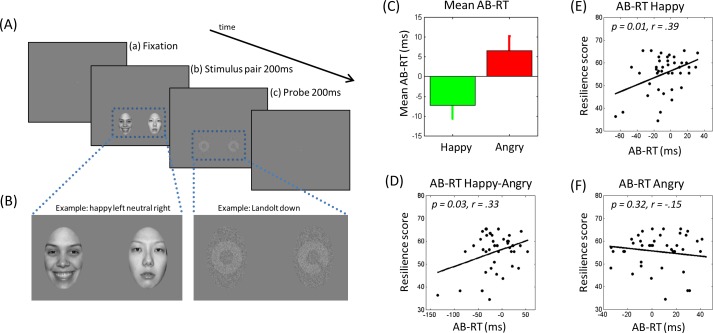
Task and behavioral results. (A, B) Modified Dot-Probe Task, (C) Mean AB reaction time scores, (D) Pearson correlations between AB happy—AB angry difference and resilience, and (E-F) between AB scores and resilience.

The experimental task was a modified DPT [13], see Fig 1A and 1B. A stimulus pair, an emotional and a neutral face, was presented simultaneously in two separate locations. A target in one of the two locations followed this, and participants had to classify this stimulus as fast as possible.I If the emotional stimulus is selectively attended, participants respond faster to the probe at the location of the emotional stimulus compared to the neutral stimulus, resulting in an AB. Target detection is generally held easy in the DPT, with typical error rates around one percent (e.g., [16]). In order to increase task sensitivity, we included a pre-DPT adjustment of individual difficulty level of probe detection. Task difficulty was controlled by adjusting the Landolt gap size using a two-up-one-down staircase procedure widely employed in psychophysics research to measure perceptual sensitivity thresholds [17].

Each trial of our DPT included (1) the presentation of a central fixation point that was constantly shown on the screen, (2) a 200 ms presentation of a face pair consisting of an emotional (i.e., happy or angry) and a neutral face, followed by (3) a 200 ms presentation of a pair of Landolt ring stimuli [18]. One of these Landolt stimuli contained an opening in the upper or lower part of the ring, the other stimulus did not contain any opening. Participants were instructed to indicate as fast and accurately as possible if the Landolt opening was located up or down by pressing one out of two response keys. Before the actual experimental task, the difficulty level was individually adjusted for each participant. A two-up-one-down parameter estimation by sequential testing staircase procedure (Garzia-Perez, 1998) was used to individually adjust difficulty levels of the DPT. The experiment (excluding the threshold task) consisted of two sessions of 192 trials each. Each session lasted approximately 10 minutes.

#### Stimuli and apparatus

The face and target stimuli were presented on a computer monitor (36.5 cm wide and 27.5 cm high) using a Windows PC computer and Cogent 2000 (http://visilab.ucl.ac.uk/cogent_2000.php) running in MATLAB (MathWorks, Natick, MA, USA). All stimuli were viewed from a 76.5 cm distance, and subtended 3.75° visual angle. The emotional stimuli consisted of 32 (i.e., happy, angry, neutral) face stimuli from the NIMSTIM set [19]. The two Landolt rings were presented at the locations of the face stimuli (see Fig 1B). All stimuli were randomized and counterbalanced; and each face appeared in all emotional conditions.

### Procedure

Testing took place during the hours of 9am and 6pm in the Laboratory for Social and Neural Systems Research (SNS-Lab) at the University of Zurich. Upon arrival, participants were instructed about the experiment and filled out an informed consent form. They completed the questionnaire measures prior to the laboratory session. After completion of the experimental task, participants were debriefed and received payment.

### Data analysis

Two participants did not complete questionnaires due to language difficulties and were removed from the analysis. Analyses of dot probe data were conducted with attention bias scores which were derived from reaction times (RT) to probes in ms. Trials with response times shorter than 200 ms (29 trials; < 1%) and longer than 1200 ms (760 trials; 4.5%) were removed (789 trials; 5.5%). RTs to probes were averaged separately for congruent (i.e., probe behind emotional stimulus) and incongruent (i.e., probe behind neutral stimulus) trials. Bias scores were caclulated separately for trials with happy faces and trials with angry faces by subtracting individual mean reaction time in congruent from incongruent trials. For both types of trials, positive bias scores indicate attention towards emotional (happy or angry, respectively) relative to neutral faces, whereas negative values indicate attention away from emotional faces.

Reaction time (RT) differences between emotional and congruent/incongruent conditions were analyzed with a two-factorial ANOVA followed by paired t-tests. To predict self-reported trait resilience, we correlated individual AB with resilience scores and calculated a linear robust regression analysis in two steps, regressing the resilience score (RS-11) on both AB scores (towards happy and towards angry faces) in the first step, and adding the interaction between the two AB scores in the second step. The interaction term was calculated by computing the product of AB towards happy and AB towards angry faces. We conducted simple slope analyses [20] for the significant interaction effect. Statistical analyses were computed using Stata 12 (StataCorp, 2011).

## Results

### Descriptives and attentional bias scores

In a 2 x 2-factorial repeated measures ANOVA with the factors emotion (happy, angry) and congruency (congruent, incongruent), we found no main effects of emotion, *F*(3, 42) = 0.42, *p* = .521, or congruency, *F*(3, 42) = 0.42, *p* = .420, but a significant interaction between emotion and congruency, *F*(3, 42) = 6.11 *p* = .018. Follow up t-tests showed that responses to congruent happy (probe after happy face; *M* = 742 ms, *SD =* 177 ms) were significantly slower than to incongruent happy trials (probe after neutral face; *M* = 734 ms, *SD* = 169 ms), *t*(42) = 2.50, *p* = .017, hence indicating an attentional bias away from happy faces in the overall sample. For angry trials, congruent, *M* = 737 ms, *SD* = 172 ms, and incongruent, *M* = 741 ms, *SD* = 175 ms, trials were not significantly different, *t*(42) = 1.60, *p* = .116.

Happy and angry AB scores were significantly different, *t*(42) = 2.48, *p* = .017 (Happy: *M* = - 8.35, *SD* = 21.80, range: -66.10–32.28; Angry: *M* = 5.76, *SD* = 23.47, range: -38.94–69.17), see Fig 1C. Importantly, there was considerable individual variability in AB scores, as necessary for the analysis of individual differences. Mean trait resilience in our sample was high, *M* = 60.88, *SD* = 9.14, range: 38–72.

### Attentional Biases and Stress Resilience

**Fig 2:**
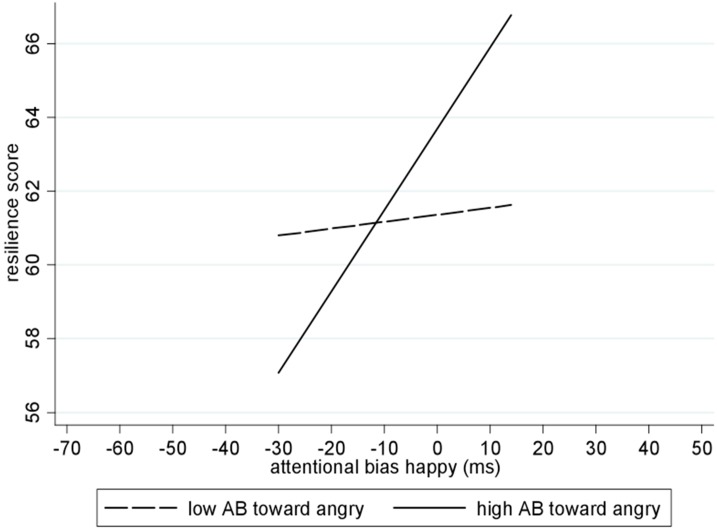
Simple slope analyses explains interaction between AB happy and AB angry scores in predicting resilience. Low AB towards angry stimuli (dashed line) represents values of 1 SD below mean, high AB towards angry stimuli (continuous line) represents values of 1 SD above the mean.

As hypothesized, greater AB towards happy, but not angry, faces was positively correlated with higher trait resilience, see Fig 1D and 1E. The first step of the robust regression showed that AB to happy faces significantly predicted higher trait resilience, *β* = .39, *t*(38) = 2.61 *p* = .013, while AB to angry faces *β* = -.01, *t*(38) = -0.10, *p* = .923 did not predict resilience, overall model: *R*^*2*^ = .15, *F*(2, 38) = 3.57, *p* = .038. When the interaction between AB towards happy and angry face stimuli was included into the model in step 2, neither AB to happy, *β* = .22, *t*(37) = 1.40, *p* = .169, nor AB to angry faces, *β* = .12, *t*(37) = 0.95, *p* = .347 alone predicted resilience, but the interaction between AB to happy and angry stimuli was significant, *β* = .38, *t*(37) = 3.28 *p* = .002. Multicollinearity was not an issue in these analyses, as correlations between AB scores were below *r* = .32. To follow up the interaction, we regressed AB for happy faces on resilience in subgroups of high and low AB towards angry faces (i.e., above and below the AB mean). As depicted in Fig 2, AB towards happy faces predicted resilience in those with high AB to angry faces (*B* = 0.19, *t* = 2.86, *p* = .007), but not in those with low AB to angry faces, (*B* = -0.01, *t* = -0.02, *p* = .985).

## Discussion

The aim of this study was to test whether an AB towards positive emotional material, alone or in interaction with an AB towards negative material, predicts trait resilience using a modified DPT. We hypothesized that an AB towards happy, and possibly an AB away from angry face stimuli, would predict resilience and explored whether the two biases interact. In line with our hypothesis, we found that an AB towards happy, but not away from angry, face stimuli predicted greater self-reported stress resilience. An AB towards angry stimuli moderated this effect. These results provide preliminary evidence for a specific relationship between stress resilience and an AB towards happy face stimuli that is modulated by AB towards angry stimuli.

Overall, we did not find an AB towards happy faces, but rather that participants tended to attend away from happy and towards neutral faces. This is in accord with prior studies and the assumption that such an AB to positive stimuli is unlikely to overrule AB to negative or neutral stimuli in magnitude in healthy participants (e.g., [21]). Importantly, however, we found considerable inter-individual variation in the extent to which participants’ attention was drawn towards positive stimuli and thus in the magnitude in which an AB towards positive emotion (or away from positive emotion) was expressed.

A first key finding was that individuals with higher AB towards positive stimuli reported greater trait resilience. The positive correlation between AB towards positive stimuli and resilience includes the interpretation that those with a bias away from positive stimuli report lower resilience, which is in line with our hypothesis. This result extends earlier findings showing that AB towards positive stimuli is not only related to adaptive stress responses and less stress-related psychopathology [2,7,22], but that it may promote stress-resilience. We found that the association was specific to an AB towards positive stimuli in our study, as there was no effect of an AB towards or away from negative stimuli on trait stress resilience. An AB towards positive stimuli may constitute a form of emotion regulation and thereby influence vulnerability to stress and psychopathology via the experience of positive emotion [23,24]. There is direct support for this assumption by experimental studies suggesting that higher AB towards positive stimuli is indeed related to more positive mood [25]. Frewen and colleagues [2] put forward the explanation that individual differences in exposure to positive stimuli may lead to subsequent differences in the magnitude to which AB towards positive stimuli are expressed, with more exposure leading to more pronounced biases. AB training studies are in accord with this assumption, as participants trained to attend to positive stimuli reported lowered levels of post-training stress [6] and stress related pathology [26]. Individual differences exist in the extent to which an AB towards positive stimuli can be trained, however, and mitigated stress responses have been found only in those participants who indeed acquired an AB towards positive stimuli in such training studies [7].

A second key finding of our study was that an AB towards negative stimuli moderated the effect of an AB towards positive stimuli on resilience, in that an AB towards positive stimuli was predictive of resilience in those individuals who also endorsed an AB towards negative stimuli. There are two possible explanations for this finding. First, only for those endorsing a tendency to be sensitive to negative stimuli might an AB towards positive stimuli be an effective and necessary means of promoting resilience, whilst those without the bias towards negative material are less in need of this potentially protective process. Second, an AB towards threat has been shown to be protective in some groups (e.g., [27,28]), and it is conceivable that such a bias, in combination with a bias towards positive material, potentiates processes related to stress-resilience. More complex attentional mechanisms may be present in resilient individuals, and it is conceivable that the two interacting biases operate at different temporal lags (see also [29]). Resilient individuals may initially orient and attend to threat and negative stimuli, but then attend to the processing of positive stimuli, which may serve as an adaptive and protective mechanism. The finding that some individuals do seem to be more prone to develop biases to both positive and negative material [11] further supports our finding of a mutual relationship between the biases. Further research will be necessary to disentangle possible mechanisms, including possible timelags at which these ABs operate, but our finding suggests that an AB towards positive stimuli may be protective, i.e., related to resilience, specifically in those individuals with a coexisting tendency to show automatic attentional responses to negative stimuli.

The current study is not without limitations. First, we used self-report questionnaires to measure trait resilience although multiple assessments of physiological and psychological functioning in naturalistic situations might be more reliable indices [30]. Second, the present sample comprised healthy control participants that were rather homogenous in trait resilience resulting in a possible ceiling effect. Third, our modified DPT indexed included a pre-threshold task to adjust the difficulty level for all participants, which may make our results less comparable with other DPT studies that exclude pre-adjustment. Fourth, we assessed attentional biases in the absence of threat or stressors. Indexing the same processes under conditions of stress might have rendered different results and should be investigated by future studies. Finally, our study is cross-sectional and thus permits causal interpretations. A prospective study is currently underway in our lab to study causal mechanisms by investigating AB towards positive emotion and its predictive vaue for later resilience in the face of stress and adversity.

Taken together, our results show that increased attention to positive emotional stimuli predicts trait resilience. While we found an overall preference for processing neutral and angry above happy faces, individual differences in AB towards positive stimuli were predictive of trait resilience in our study. An AB towards positive stimuli may thus not constitute a “default” state endorsed by all individuals alike, but individual differences in such an AB are associated with enhanced ability to adapt to stressful situations. The finding also suggest that AB towards positive and negative stimuli interact in influencing resilience, with an AB to positive stimuli being protective in those also endorsing an AB to negative stimuli. This has implications for the development of stress prevention programs and for improving treatment of stress-related disorders. Manipulating types of stimuli individuals attend to may help decrease vulnerability to stress-related psychopathology and increase resilience. Our results suggest that increasing the tendency to attend to emotionally positive stimuli could render individuals, specifically those who are also sensitive to negative emotional stimuli, more resilient.
